# Development and validation of a fall health literacy scale for Chinese hospitals from the perspective of older adults

**DOI:** 10.3389/fpubh.2025.1675579

**Published:** 2025-12-03

**Authors:** Tianxin Miao, Ke Chen, Dianli Han, Yingna Zhao, Liran Duan, Lan Zhang, Ying Yao

**Affiliations:** 1 Tianjin Medical University General Hospital, Tianjin, China; 2 Department of Emergency Medicine, General Hospital of Tianjin Medical University, Tianjin, China

**Keywords:** older adults, falls, health literacy, scale, psychometrics, hospitalized

## Abstract

**Background and aim:**

Health literacy plays an important role in assessing patients’ ability to receive information about falls and in educating patients about their health. Understanding the level of fall health literacy of hospitalized older adults is essential for implementing fall prevention interventions, but tools for assessing the fall health literacy of hospitalized older patients are lacking. The aim of this study was to develop and validate an instrument for assessing fall health literacy among hospitalized older adults in China.

**Methods:**

The Fall Health Literacy Scale for Hospitalized older adults was developed in two phases. In the first stage, dimensions and items were generated through a systematic literature review, semistructured interviews, and expert consultation. In the second phase, a cross-sectional study was conducted on 450 hospitalized older adults aged 60 years and above in two tertiary hospitals in Tianjin from March to July 2025 to test the reliability and validity of the items.

**Results:**

The scale comprises 30 items across five dimensions: cognition of fall information, transformation of fall information, implementation of fall information, knowledge of fall information, and personal attitudes and resource factors. This scale demonstrated excellent reliability, with a Cronbach’s *α* of 0.926, a split-half reliability of 0.884, and a test–retest reliability of 0.897. Furthermore, the results of the confirmatory factor analysis confirmed the good construct validity and reliability of the scale.

**Conclusion:**

The scale has good reliability and validity and is a tool for evaluating fall health literacy from the perspective of older adults; additionally, it provides a new perspective for reducing the incidence of falls in China.

**Clinical trial registration:**

https://www.chictr.org.cn/showprojEN.html?proj=273509.

## 1 Introduction

Inpatient falls are the most common safety incidents, and they not only cause physical injury to patients but also impose very large financial burdens (1, 2). Studies have shown that the incidence of falls among older adults in China is 1.5 times greater than that in community settings (3). However, most falls are not actually accidents and are not an inevitable consequence of aging. Instead, falls are the result of a complex interplay of multiple intrinsic (patient-related) and extrinsic (environmentally related) risk factors coupled with risky behaviors (4). An unfamiliar hospital environment, coupled with acute physical and/or psychiatric illness, further increases the risk of falls in hospitalized older adults (5).

Research on fall prevention in hospitalized older patients has evolved from focusing on external factors [e.g., environmental modification (6), nurse staffing (7), and nurse risk assessment, followed by targeted health education (8, 9)] to exploring intrinsic factors [including patient psychology (10), perceptions of falls (11, 12), and the willingness to participate in fall prevention (13)]. The results show that the importance of patients for preventing falls is gradually being recognized (14); however, this recognition is limited to psychological research on patient falls and does not further explore the ability of patients to prevent falls.

In clinical practice, the prevention of falls in hospitalized older adults mainly depends on fall risk assessment to identify high-risk patients, followed by targeted health education. There are two limitations to this method: first, the risk assessment of patients relies mainly on traditional tools such as the Morse Scale and the St. Thomas Risk Assessment Tool in Falling Older Adults, which can identify high-risk groups but fail to assess the cognitive and behavioral ability of patients to prevent falls from the perspective of patients (5, 6). Second, targeted health education should also assess whether patients have the ability to understand and apply the information (15, 16).

Health literacy refers to an individual’s ability to access and understand basic information and services and to use them to make correct decisions to maintain and promote his or her own health, and it is related to age, gender, education level, etc. There are disparities across populations and settings, and these disparities affect how easily people can develop and use health literacy and whether they have access to high-quality, trustworthy information and services (17). From a health literacy perspective, it is critical to assess the ability of hospitalized older adults to obtain, understand, assess, and utilize fall-related information so that targeted interventions can be implemented to improve individual fall prevention capabilities (18). A growing body of research has highlighted the critical role of health literacy in fall prevention (19–21). Currently, while some studies (22–24) have applied general health literacy assessment tools to fall-related populations, few instruments have been designed specifically to assess fall-related health literacy. Lim et al. (25) developed a fall-related health literacy assessment tool primarily targeting older adults living in the community. Although this scale demonstrates general applicability, there are significant differences between community and hospital settings. As a result, their tool lacks specificity for hospitalized older patients, highlighting the need for further research in this area.

The aim of this study is to develop a fall-specific health literacy scale for hospitalized older adults, the China Health Literacy Scale for Falls in Hospitalized Older Adults (CFHLS), and to test its psychometric properties.

## 2 Method

The research process was divided into two stages. First, scale development involved a literature review, semistructured interviews, and expert consultation. Second, the scale was validated in a cross-sectional study to evaluate its reliability and validity.

### 2.1 Scale development

#### 2.1.1 Framework for the development of the CFHLS

A conceptual model of the health literacy of the Chinese population was generated by Liu et al., who summarized and categorized previous health literacy-related research into four parts: antecedents, cores, intermediaries and results. Liu et al. argued that health literacy is not only the ability to obtain, understand, evaluate, make decisions and apply health information but also affects individuals’ ability to maintain knowledge, personal personality and resources; thus, several other factors should be considered when health literacy is assessed (26). This health literacy model integrates clinical and public health perspectives and adds the role of health service providers in promoting health literacy, which is relatively comprehensive. Therefore, this study uses this model to guide the development of the CFHLS.

#### 2.1.2 Literature search

This study used a systematic retrieval strategy to obtain Chinese and English-language research, guidelines, evidence summaries, evaluation criteria and evaluation tools on the health literacy of hospitalized older adults from the following databases and platforms: English-language databases—PubMed, Web of Science, the Cochrane Library, and Embase; Chinese databases—the China Knowledge Network Infrastructure (CNKI), Wanfang, and SinoMed (China Biomedical Literature Database); and other resources—Guidelines International Network (GIN), the World Health Organization (WHO), the National Health Commission, Medical Pulse and NICE. Search strategy index words were combined with free words: medical keywords (MeSH) and database-specific goals (for example, older adults, falls, and health literacy, scales) were the core, including free terms. As the search formula logic, a Boolean operator was used to link search terms (OR) of the same concept and to combine different concepts (AND). The time frame was from the establishment of the database to March 2025. Non-Chinese and non-English-language research, works whose full text could not be obtained, and nonacademic content (such as news and editorials) were excluded. Based on the conceptual model of the health literacy of the Chinese population, a pool of 45 items for the CFHLS was initially established, including four dimensions (fall information-related ability, fall knowledge, fall prevention and maintenance ability, and individuals).

#### 2.1.3 Semistructured interviews

The purposive sampling method was used to select 16 inpatients aged 60 and above in a tertiary hospital in Tianjin for interviews. The interview outline addressed (1) falls: How do you feel about the issue of falls? What kind of people do you think need fall prevention? Have you ever experienced a fall? Do you think you need fall prevention? Why? Where do you seek help when you feel you need to prevent falls? Why? (2) It also addressed falls in the hospital: During your hospitalization, do you think that you are at risk for falls? Why? Have you ever experienced a fall in hospital? Can you describe the circumstances? (3) Furthermore, it addressed fall health literacy: Assuming that you had a fall, would you know what to do? Where do you usually obtain information about falls? Please describe in detail the information that you obtain about falls. Which of this information that you obtained did you use during your hospitalization? Did you experience any difficulties when you used this information? In accordance with the interviewees’ answers, we summarized, refined the common parts, supplemented the scale item pool, and added 2 personal dimension items: “I can summarize the lessons of past falls and am able to deal with the same situation correctly when it happens again” and “I am a very independent person and do not like to trouble others, especially for daily activities such as going to the toilet and going to the floor”.

#### 2.1.4 Delphi expert consultation

A total of 15 experts, representing eleven universities and hospitals in China, were invited to evaluate the importance of the project. Two rounds of consultation were conducted online via email platforms. Notably, all 15 experts participated, resulting in a 100.0% response rate. Furthermore, the coordination factor achieved was between 0.141 and 0.275, indicating a statistically significant result at the 0.05 level (*p* < 0.05). Based on expert opinions and group discussions, we made the following changes: (1) item optimization; (2) restructuring: adjusting the order of dimensions; and (3) item reduction—duplicate items were removed from the initial pool of 47 scale items, resulting in a scale with 32 items (see Supplementary material for details). We subsequently recruited 15 hospitalized older patients for a pilot study to adjust the wording and ensure the comprehensibility of the survey.

### 2.2 Scale validation

#### 2.2.1 Sample and setting

In general, the sample size needs to be 5 to 10 times the number of metrics (items) measured for more rigorous testing and evaluation of the performance of the scale. The initial version of the scale developed in this study contained 32 items, and the sample size for exploratory factor analysis and confirmatory factor analysis should not be less than 200; considering the possibility of invalid questionnaires, an additional 10% of the sample size was needed, i.e., 320 × 1.1 = 352; thus, the sample size should not be less than 400 cases (27). In this study, a convenient sampling method was used to recruit hospitalized older adults from two tertiary grade A hospitals in Tianjin, China, targeting departments with a high volume of older patients, namely, emergency medicine, endocrinology, neurology, and geriatrics. The inclusion criteria were as follows: (1) inpatients aged ≥ 60 years; (2) hospitalized for more than 24 h; and (3) voluntary participation in the study and signing of the informed consent form. The exclusion criteria were as follows: (1) cognitive impairment; (2) loss of consciousness, such as coma, drowsiness or an inability to clearly express one’s wishes; (3) serious illness or unstable vital signs; and (4) participation in other related research at the same time.

#### 2.2.2 Questionnaires

The general information questionnaire was developed by the authors and encompassed gender, age, household registration, literacy, and other fundamental information. The survey utilized a formal scale comprising 30 items across four domains: the ability to recognize and transform information from falls, the ability to act on information on falls, the degree of fall knowledge mastery, and personal traits. With the exception of Item 21 (“I know that the following factors increase my risk of falling during hospitalization”) and Item 23 (“I know that the following measures can prevent a fall during my hospitalization”), the scale used a 5-point Likert scale as a scoring scale, and the total score was converted such that 0–2, 3–4, 5–6, 7–8, and 9–10 were 1, 2, 3, 4, and 5 points, respectively. Item 27 (“In the hospital, I do not think I’m in danger of falling”) was reverse scored. The remaining items were scored positively, with a score of 1 representing “strongly disagree” and a score of 5 representing “strongly agree,” where higher scores indicate a higher level of health literacy in terms of patient falls.

#### 2.2.3 Data collection

An online questionnaire was administered via Questionnaire Star, and all 450 hospitalized older adults were informed of the purpose, significance, and content of the study, as well as guidelines and precautions for completion prior to the survey. The patients were also informed that their answers and scores were confidential and anonymous. The participants read an electronic version of the informed consent form, agreed to participate in a self-administered electronic questionnaire, and took the online survey. If the patients had questions while completing the questionnaire, the person in charge at the site answered the questions to them in a reasonable manner and with uniform instructions. A total of 450 questionnaires were received online, and after careful checking, the validity of the questionnaires was 100%.

#### 2.2.4 Analyses

Raw and coded data were exported via Questionnaire Star, followed by conversion and statistical analysis via PASW 18.0 and AMOS 23.0. Count data were described via frequencies and percentages (*N*, %), whereas normally distributed data were described via the mean ± standard deviation ( $\bar{x}\pm s$ ). The reliability of the expert inquiry was assessed via indicators such as the expert familiarity coefficient, Kendall’s coefficient of concordance, and the authority coefficient (28). Bartlett’s test of Sphericity and the sampling adequacy test (Kaiser–Meyer–Olkin, KMO) were used to assess whether factor analysis was appropriate, with a *p* value of < 0.05 for the Bartlett test and a KMO value of > 0.8 considered the criteria for appropriate factor analysis (29, 30). Item analysis [analysis of the discrete trend method, critical ratio method, correlation coefficient method and Cronbach’s *α* method (evaluation of whether Cronbach’s α increases after any item on the scale is deleted)] was used to accurately screen the scale items (31, 32). The structural validity of the scale was evaluated via exploratory factor analysis, confirmatory factor analysis, convergent validity, discriminant validity, the correlation between factor scores, and the Pearson correlation coefficient between each factor score and the total scale score (33, 34).

### 2.3 Quality control

To ensure data integrity throughout the research process, the following measures were implemented.

#### 2.3.1 Semistructured interviews

All interviewers received formal training. Before the interview, the participants were informed of the study’s purpose and procedures and the use of recording; written informed consent was obtained. The interviews were conducted in a quiet, private conference room by two researchers: one led the discussion using the outline, and the other recorded and supplemented, with both interviewers avoiding leading language. Verbatim transcription was completed within 24 h, and the transcripts were returned to the participants for checking.

#### 2.3.2 Delphi expert consultation

Experts were selected based on strict criteria. They were fully briefed on the study’s purpose before the process. The consultation was continued until the expert opinions reached consistency. Expert positivity, authority, and the degree of opinion coordination were calculated to ensure the credibility of the results.

#### 2.3.3 Data collection and analysis

To minimize selection bias, subjects were recruited in strict accordance with the inclusion and exclusion criteria. The investigators were uniformly trained to standardize data collection and prevent information bias. The data were logically checked for inconsistencies. Statistical methods were selected and applied in consultation with statistical experts to ensure appropriateness and accurate interpretation.

### 2.4 Ethical considerations

This study adhered to the Declaration of Helsinkiethical principles and was approved by the Ethics Committee of Tianjin Medical University General Hospital on January 23, 2025 (approval number: IRB2025-YX-039-01).

## 3 Results

### 3.1 General patient information

From March to July 2025, 450 hospitalized older adults were recruited from two tertiary grade A hospitals in Tianjin, China, via convenience sampling, targeting departments with a high volume of older patients, namely, emergency medicine, endocrinology, neurology, and geriatrics: 250 were used for exploratory factor analysis, and 200 samples were used to verify the fit of the model. Of these, 250 (55.6%) were male, and 200 (44.4%) were female. In terms of age, 289 (64.2%) were aged between 69 and 74 years, 149 (33.1%) were aged between 75 and 89 years, and 12 (2.7%) were aged 90 years and older, with an average age of 72.30 ± 8.03 years. See Table 1 for details.

**Table 1:** General information of patients (*n* = 450).

Event		Number	Percentage (%)
Gender	Male	250	55.6
	Female	200	44.4
Age	60–74 years	289	64.2
	75–89 years	149	33.1
	≥90 years	12	2.7
Education level	Uneducated	34	7.6
	Elementary school	91	20.2
	Junior high school	123	27.3
	High school/secondary school	128	28.4
	College degree or above	74	16.4
Marital status	Unmarried	3	0.7
	Married	367	81.6
	Divorce	3	0.7
	Widowed	77	17.1
Number of children	0	6	1.3
	1	235	52.2
	2	146	32.4
	≥3	63	14.0
Medical payment methods	Urban medical insurance	328	72.9
	New rural cooperative medical insurance	120	26.7
	Self-Payment	2	0.04
Place of residence	Town	354	78.7
	Countryside	96	21.3
Monthly income per capita	<4,000 RMB	137	30.4
	4,000–8,000 RMB	239	53.1
	8,000–12,000 RMB	69	15.3
	>12,000 RMB	5	1.1
Has there been a fall in the last year	Yes	96	21.3
	No	354	78.7
Risk of falls	Low	251	55.8
	Medium	170	37.8
	High	29	6.4

### 3.2 Content validity

According to the established criteria, the content validity of a scale can be considered adequate when the I-CVI is not less than 0.78 and the S-CVI is not less than 0.90 (28). The 15 experts were from different geographical regions. All 15 specialists had at least a bachelor’s degree, and most of them had qualifications at the level of associate professor or associate chief nurse or equivalent. In addition, all 15 specialists had accumulated at least 10 years of theoretical knowledge and practical experience in geriatric nursing, nursing education, clinical nursing, nursing administration, patient safety management, and health education. They were recognized as leaders in their respective fields. The results of the content validity analysis revealed that the CFHLS exhibited excellent content validity, with an I-CVI ranging from 0.800 to 1.000 and an S-CVI ranging from 0.938.

### 3.3 Project analysis

The results of the discrete trend analysis demonstrate that the standard deviation of the scores for each item ranges from 1.000 to 1.391, indicating that the discrete trends of each item are reliable. The correlation coefficient between the item score and the total score is the correlation coefficient calculated between the item score and the total scale score. Items with insignificant correlation coefficients and those with correlation coefficients less than 0.4 are then excluded (34). In this study, two items with correlation coefficients < 0.4 with respect to the total score were excluded after discussion, and the remaining items with correlation coefficients between 0.434 and 0.696 (*p* < 0.01) were eligible. The Cronbach’s *α* coefficient of the scale was 0.926, with no significant increase after any individual items were deleted. The critical ratio method is employed to calculate the subject’s score on the scale item, which is ranked from high to low, and the top and bottom 27% are selected as the high and low groups, respectively (35). The significance of the difference in scores between the high and low groups is then compared via an independent sample t test. If *p* < 0.05, the item is retained because it has good discriminant validity; otherwise, it is deleted (28). The study employed independent samples t tests for the analysis of the data, and the results indicated that the differences in the mean scores of each item were statistically significant (*p* < 0.01), thereby suggesting that all the items in the scale possessed adequate discrimination and should be retained.

### 3.4 Internal consistency and reliability evaluation

Cronbach’s *α*, split-half reliability, and test–retest reliability were used to assess the internal consistency and reliability of the scales. Alpha coefficients with values of 0.7–0.8 were considered satisfactory, values of 0.8–0.9 were considered good, and values greater than 0.9 were considered excellent. Cronbach’s α coefficients > 0.60 for the subscales were considered to be at the desired level of reliability (34). In the present study, the Cronbach’s α coefficient for the overall scale was 0.926, and the split-half reliability was 0.884. The Cronbach’s α coefficients for the five factors ranged from 0.834 to 0.937, and the split-half reliabilities ranged from 0.832 to 0.938 for the same five factors. These results indicate that the scale and its five factors have satisfactory reliability and internal consistency. In addition, retesting the scale after 2 weeks with 20 older adults revealed a test–retest reliability of 0.884 for the overall scale and between 0.845 and 0.942 for the five factors (36). These findings suggest that the results of the two reliability tests are relatively consistent with minimal error and that the scale exhibits high measurement stability over longer periods of time or repeated use (see Table 2).

**Table 2:** Internal consistency and retest reliability of the scale (*N* = 250).

Dimension	Item count (of a consignment)	Cronbach’s α coefficient (*n* = 250)	Split-half reliability (*n* = 250)	Retest reliability (*n* = 20)
Total	30	0.926	0.884	0.897
Cognition	8	0.919	0.916	0.837
Transformation	3	0.834	0.832	0.894
Implementation	9	0.937	0.938	0.906
Knowledge	6	0.927	0.924	0.799
Personal	4	0.900	0.882	0.826

### 3.5 Validity analysis

#### 3.5.1 Structural validity

##### 3.5.1.1 Exploratory factor analysis

The suitability for factor analysis was assessed via Bartlett’s test of sphericity and the KMO test. A *p* value of < 0.05 for the Bartlett test and a KMO value of > 0.8 were considered the criteria for suitability for factor analysis (37). In accordance with the criterion of an eigenvalue > 1.0, maximum variance rotation was applied to these factors, and items with loadings greater than or equal to 0.40 were considered independent factors. The analysis yielded a 5-factor structure, and the factors were designated implementation, cognition, knowledge, personal, and transformation. The cumulative variance contribution rate was determined to be 70.194%, which exceeded the 50% pass standard (36). See Supplementary Table S1 for details.

##### 3.5.1.2 Confirmatory factor analysis

**Figure 1:**
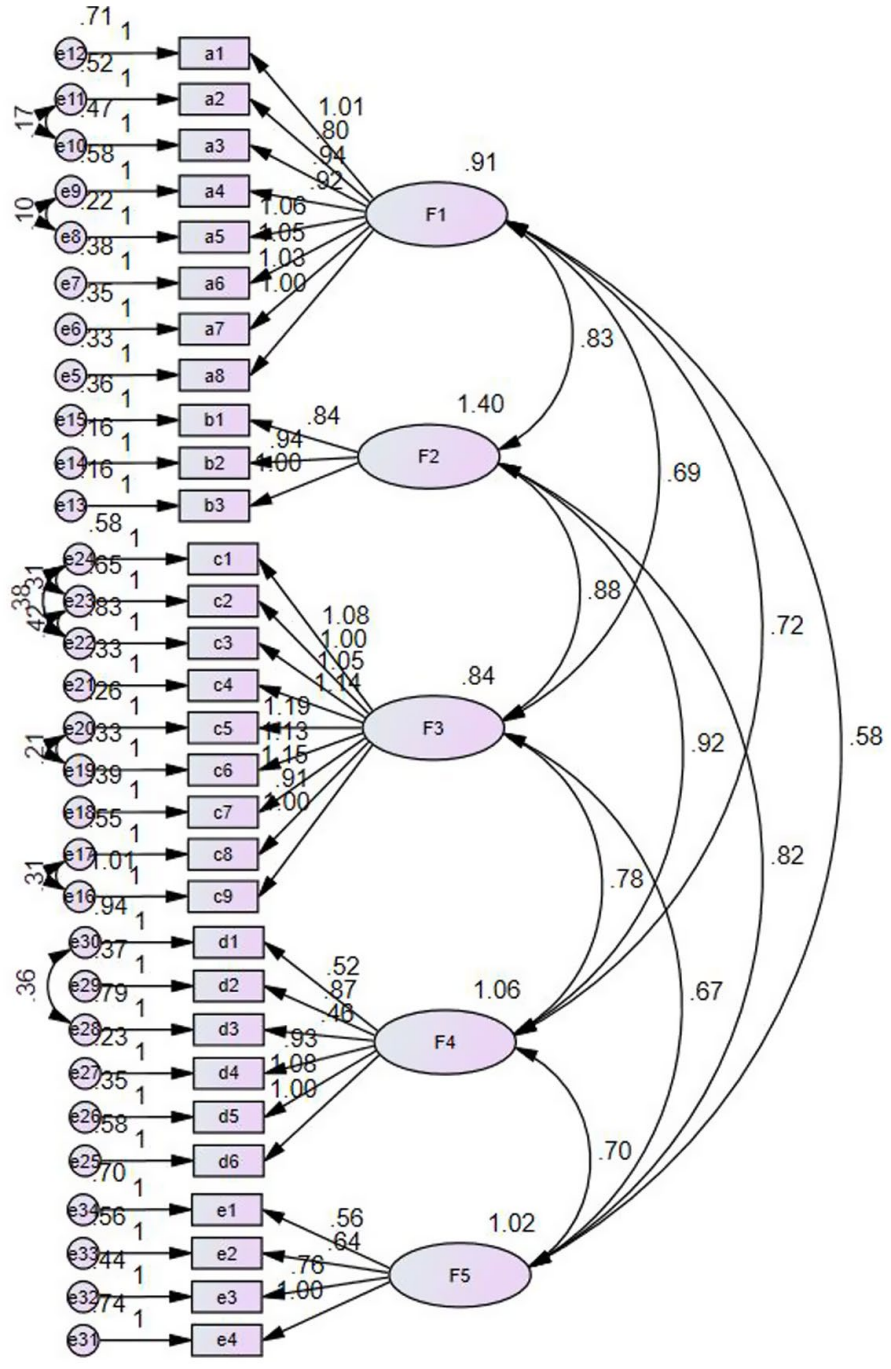
Standardized path coefficients for the final model of the validation factor analysis (*N* = 200).

The results of the confirmatory factor analysis were corrected for MI based on the conceptual model. Data not corrected for MI are shown in Supplementary Table S2. The results revealed that the chi-square statistic (χ^2^/*df*) = 1.852, the root mean square error of approximation (RMSEA) = 0.065, and the standardized root mean square residual (RMR) = 0.069, which were within the acceptable thresholds. Furthermore, the CFI, TLI, and IFI were greater than 0.9, and the NFI was greater than 0.8, which were all over the acceptable thresholds, indicating that the model was well fitted (38) (see Table 3). The five dimensions—cognition, transformation, implementation, knowledge and personal—are represented by F1, F2, F3, F4 and F5, respectively (see Figure 1).

**Table 3:** Model fit indicators (*N* = 200).

Commonly used indicators	χ^2^	χ^2^/*df*	GFI	RMSEA	RMR	CFI	NFI	TLI	AGFI	IFI	RMSEA 90% CI
Judgement criteria	-	<3	>0.9	<0.1	<0.05	>0.9	>0.9	>0.9	>0.9	>0.9	-
Value	716.855	1.852	0.811	0.065	0.069	0.939	0.878	0.932	0.773	0.940	0.058–0.073

##### 3.5.1.3 Convergent and discriminant validity

Typically, high convergent and discriminant validity is indicated if the AVE (average variance extracted) > 0.5 and CR (composite reliability) > 0.7 (33). In addition, the square root of the coefficient should be greater than the correlation coefficient between factors (39). In this study, the CR for each dimension was 0.940, 0.939, 0.942, 0.878, and 0.778. The AVE was 0.663, 0.834, 0.646, 0.560, and 0.471. And the square roots of all AVEs were greater than the correlation coefficient between factors, which suggests that the scale has very good convergent and discriminant validity (see Supplementary Tables S4, S5).

#### 3.5.2 Correlations between factor scores and scale totals

The Pearson correlation coefficient between the 5 factor scores ranges from 0.521 to 0.787, and the correlation coefficient between the 5 factor scores and the total score of the scale ranges from 0.715 to 0.935 (*p* < 0.01). The evidence indicates a clear correlation between the items of the Fall health literacy Scale for hospitalized older adults and their common factors. In addition, the correlation between factor scoring and overall evaluation is relatively strong (see Supplementary Table S6).

## 4 Discussion

With the increasing specialization of health literacy research, assessment tools in subfields such as oral, mental, and e-health literacy (43–45) have become increasingly systematic and specialized. However, in the critical area of fall health literacy, existing assessments still largely rely on generic health literacy tools that are not specific to the issue of “falls” (22–24). To some extent, this has hindered the theoretical development of fall health literacy as an independent research concept. Li et al. (20) demonstrated that inadequate health literacy may indirectly increase the fall risk among community-dwelling older adults by influencing health behaviors, physical function, and gait speed, while improving health literacy can help reduce fall incidence. Nevertheless, research on the relationship between health literacy and fall incidence among hospitalized older patients remains limited, largely because of the absence of effective tools for assessing fall health literacy in this specific population.

This study successfully developed and validated a fall health literacy scale specifically for hospitalized older adults in China, which was constructed based on a health literacy conceptual model for the Chinese population and consists of 30 items. It demonstrates good psychometric properties and strong comprehensiveness and is well suited to the target population.

After two potentially redundant items were removed, the scale showed reasonable internal consistency. Exploratory factor analysis generated a factor model indicating an appropriate overall distribution of dimensions, with a five-factor structure that accurately captured the characteristics of fall health literacy among hospitalized older adults. The analysis identified five core components: cognition of fall information, transformation of fall information, implementation of fall information, knowledge of fall information, and personal attitudes and resource factors. Unlike existing health literacy conceptual frameworks (40, 41), the model used in this study not only reflects the traditional skill- or competency-based view of health literacy but also innovatively incorporates additional dimensions such as other abilities, knowledge, personality traits and personal resources. By treating health literacy as an outcome of the interaction between patients, healthcare providers, and the healthcare system, this model addresses the limitations of previous studies that overemphasized health literacy as an individual skill, and it highlights the important role of the healthcare system and healthcare providers in promoting population health literacy (26).

The study provided evidence for the reliability and validity of the tool. The internal consistency, as measured by Cronbach’s *α*, ranged from 0.834 to 0.937 for each dimension and was 0.926 for the total scale, indicating high stability and satisfactory homogeneity over time (24). Correlational analysis between dimensions revealed that all correlation coefficients fell within a reasonably acceptable range (0.434–0.696) (46). The test–retest reliability results demonstrated good stability of the scale. Confirmatory factor analysis indicated an acceptable model fit and good construct validity (47). Although the adjusted goodness-of-fit index (AGFI) did not reach the desired threshold, possibly owing to its sensitivity to model complexity, this outcome did not compromise the overall model fit (48). Future research should involve a larger sample size to refine the scale’s items and structure, thereby further enhancing its psychometric properties. Furthermore, the scale can be completed in just 6–8 min, ensuring its feasibility for both population screening and scientific research applications.

In recent years, Lim et al. (25) developed the Falls Health Literacy Scale (FHLS), which comprises both subjective and objective components. The subjective component focuses on core competencies such as accessing, understanding, appraising, and applying information related to falls, whereas the objective component uses an illustrated booklet to simultaneously assess knowledge and provide education. However, when Peng et al. (42) adapted this scale for use in China, the objective section was omitted. Although the Chinese version demonstrated good reliability and validity in screening hospitalized older adults for general health literacy inadequacy, it cannot accurately identify specific gaps in fall-related knowledge. Furthermore, although it shows good psychometric properties in this population, the scale was not originally designed for the inpatient setting. Its content does not distinguish between hospital and community environments, and since the environment is a key influencing factor for health literacy, this likely introduces measurement bias. These limitations constrain the scale’s utility in guiding targeted health education interventions in clinical practice. Meanwhile, against the backdrop of China’s rapidly aging population, falls among older adults have emerged as a critical public health issue. In response, the State Council explicitly identified “strengthening the prevention and control of disability caused by falls in the older adults” as a key initiative in the National Disability Prevention Action Plan (2021–2025) (49), emphasizing the goal of “enhancing citizens’ awareness and capacity for disability prevention.” In line with this policy direction, there is a pressing need to develop a context-specific and practically oriented assessment tool for fall health literacy. Therefore, unlike the Peng et al., the present study developed a self-reported fall health literacy scale specifically designed for hospitalized older adults. The scale covers the entire process from prevention to post-fall management, with a particular focus on risk identification, preventive behaviors, and patient–provider collaboration within the hospital setting. And in the knowledge assessment section, based on existing guidelines and validated knowledge instruments (50, 51), the scale includes 10 essential elements related to risk recognition and fall prevention, allowing patients to indicate their understanding through a checklist. Additionally, knowledge related to responding to a fall is organized in a logical sequence—before, during, and after the fall—to align with natural cognitive processes, supporting not only rapid clinical assessments, but also targeted clinical interventions based on assessment results.

The fall health literacy assessment tool used in this study is a self-reported measure. Compared with the internationally commonly used cognitive testing health literacy tools, the self-report design has unique advantages: it can cover multiple dimensions of health literacy and more directly reflect the cognitive status of participants in daily situations and their impact. However, the self-reporting method also has certain limitations. Lee et al. (52) reported in a study of the Taiwanese population that male respondents clearly tended to overestimate in self-reported questions. This result shows that the two methods of cognitive testing and self-reporting have their own advantages and disadvantages, and they need to be weighed in combination with research objectives when applied.

Based on the considerations above, when this scale is used in the future, full attention should be paid to the measurement deviations that may be caused by the self-reporting method. In actual clinical applications, this scale should be used as a first-stage tool for admission screening, and it should be integrated into the hospitalization assessment system to quickly and efficiently identify older adults with low health literacy. The system can automatically identify high-risk groups based on the scoring results and then be combined with other clinical fall risk assessment tools to form a complementary mechanism combining self-assessment and other assessments to construct an efficient and accurate closed-loop management process for fall prevention.

## 5 Limitations

First, the length of the scale may pose challenges for its widespread clinical application. Although we have condensed the instrument to 30 items, its practicality could still be impacted by the fast-paced nature of clinical environments, the specific needs of older adults, and the heavy workloads of healthcare staff. To enhance feasibility, future efforts should focus on developing a short-form version for rapid screening or considering embedding the scale in the form of an electronic form into hospital information systems to optimize assessment efficiency. Second, this scale is based on the current situation of the hospital environment in China; thus, its cross-cultural adaptability should be further tested in future research.

Third, owing to the convenience sampling method, the study sample was exclusively from two hospitals in Tianjin, China, and the representativeness of the results may be limited; thus, a multicenter study across different levels of healthcare systems, departments, and populations is expected to further evaluate the measurement invariance and longitudinal reliability of the scale.

## 6 Conclusion

The Fall Health Literacy Scale for Chinese Hospitalized Older Adults developed in this study contains 5 components and 30 items with acceptable reliability and validity. It provides an effective tool for the cognition, transformation, and executive ability assessment of fall information in older adults in China and the setting of targeted interventions.

## Data Availability

The raw data supporting the conclusions of this article will be made available by the authors, without undue reservation.
