# Supplementary material for: Development and validation of a fall health literacy scale for Chinese hospitals from the perspective of older adults
Source: Front Public Health. 2025 Dec 3;13:1675579. doi: 10.3389/fpubh.2025.1675579 (PMC12708289; doi:10.3389/fpubh.2025.1675579)
Supplement: Supplementary file 1 [file Data_Sheet_1.PDF]

Delphi expert consultation

Design expert consultation questionnaire

The expert consultation questionnaire was designed based on the initial version of the scale. The questionnaire consisted of three parts: (1) instructions for respondents, which mainly included the background and purpose of the study, clarification of relevant concepts, and guidelines for completing the questionnaire; (2) the basic information about the expert, incorporating factors such as age, title, academic background, position, field of work, and professional experience; a form for assessing the expert's familiarity; and a form for self-assessment of the basis of the expert's judgments; and (3) a form for consulting on the scale's indicators, which included Indicator Importance Evaluation (Likert 4-point scale: 1=not important, 4=very important). Each item has a field for post-setup modification of comments (add/delete/modify advice).

Expert selection criteria

(1) Bachelor's degree or above (2) Associate professor/associate chief nurse or above (3) 10 years or above in related fields (clinical nursing, nursing education, nursing management, patient safety management), with representativeness and authority within the discipline (4) Informed consent and full understanding of the study content, as well as the ability to comprehensively provide professional advice and suggestions.(5) From different geographical regions.

Item screening criteria

The threshold for screening scale items is a mean score of at least 3.5 and a coefficient of variation of no more than 0.25. Following consideration of the consensus of experts and group discussions, the scale items were determined using the most appropriate method.

Table S1 Rotated Component Matrix for Factor Analysis of the Falls Health Literacy Scale for Hospitalized Elderly Patients (N = 250)

items	Ingredient				
	Implementation	Cognition	Knowledge	Personal	Transformation
1.I can access fall information through a variety of sources such as fall warning signs, electronic devices and hospital lectures.	0.095	**0.797**	0.139	0.095	0.031
2.I can understand getting information about falls.	0.100	**0.790**	0.068	0.062	0.154
3.I can accurately describe to others the personal information I have obtained.	0.139	**0.782**	0.105	0.088	0.167
4.I can determine which fall information is credible.	0.181	**0.772**	0.204	0.044	0.082
5.I can determine if the information I receive about falls is appropriate for me.	0.098	**0.781**	0.052	-0.032	0.044
6.I can analyze my own needs for fall prevention.	0.083	**0.759**	0.149	0.037	0.041
7.I can compare different fall information according to my own needs.	0.140	**0.765**	0.110	0.058	0.088
8.I can choose the personal information that suits me.	0.096	**0.757**	0.200	0.079	0.012
9.I can prevent myself from falling when I haven't fallen.	0.181	0.129	0.215	0.167	**0.798**
10.I can respond correctly to avoid injury in the event of a fall.	0.103	0.156	0.142	0.233	**0.789**
11.I can take the right steps to avoid aggravating the injury after a fall.	0.094	0.175	0.122	0.129	**0.830**
12.I can share my experience in fall prevention with others.	**0.822**	0.075	0.198	0.100	-0.002
13.I can explain falls to the person in the ward when needed.	**0.791**	0.168	0.008	0.074	0.015
14.I can ask the reason, when I don't understand the fall information from the medical staff.	**0.767**	0.052	0.046	0.049	0.068
15.I can objectively evaluate my physical and psychological advantages and disadvantages in preventing falls.	**0.777**	0.169	0.170	0.150	0.080

items	Ingredient				
	Implementation	Cognition	Knowledge	Personal	Transformation
16.I can reflect on whether I strictly follow the recommendations of the medical staff to prevent falls.	**0.815**	0.164	0.044	0.104	0.109
17.I can reflect and evaluate my inner thoughts: such as the attitude of preventing falls, the fear of falling.	**0.811**	0.079	0.160	0.040	0.003
18.I can summarize the lessons learned from my own or others' past falls and respond correctly when the same situation occurs again.	**0.783**	0.125	0.142	0.123	0.063
19.I can adapt quickly to my new inpatient environment.	**0.798**	0.131	0.006	0.109	0.087
20.I can set an exercise goal and work towards completing it to improve my balance to prevent falls.	**0.783**	0.064	0.164	0.071	0.148
21.I know that the following factors increase my risk of falling during hospitalization.	0.094	0.203	**0.844**	0.057	0.019
22.I know the exact locations in the hospital where falls are likely to occur.	0.082	0.187	**0.805**	0.036	0.151
23.I know that the following measures can prevent a fall during my hospitalization.	0.106	0.14	**0.858**	0.026	0.104
24.When I feel dizzy, I know to look for something to support me at the first opportunity.	0.110	0.177	**0.795**	0.015	0.128
25.When I have a fall, I know that I should protect my head first.	0.187	0.104	**0.826**	0.091	0.161
26.After falling, I knew I should stay where I was, call the medical staff, and wait for help.	0.178	0.123	**0.815**	0.108	0.003
27.In the hospital, I don't think I'm in danger of falling.	0.134	0.065	0.057	**0.806**	0.145
28.In the hospital, I think it's important to prevent falls.	0.175	0.073	0.055	**0.850**	0.171
29.I can find someone with whom I could talk about my concerns about falls.	0.150	0.065	0.066	**0.907**	0.104
30.I can afford a person who can help me prevent falls. (e.g., nurse practitioner, health care provider, etc.)	0.117	0.101	0.086	**0.834**	0.089

Extraction method: principal component analysis. Rotation method: Kaiser normalized maximum variance method. Rotation has converged after 6 iterations.

Table S2 Model fit indicators (N = 200) Not corrected by MI

Commonly Used Indicators	χ^2	χ^2/df	GFI	RMSEA	RMR	CFI	NFI	TLI	AGFI	IFI	RMSEA 90% CI
Judgement Criteria	-	<3	>0.9	<0.1	<0.05	>0.9	>0.9	>0.9	>0.9	>0.9	-
Value	1084.858	2.746	0.714	0.094	0.079	0.873	0.815	0.860	0.663	0.874	0.087-0.100

Convergent and discriminant validity

Convergent validity means that items measuring the same underlying trait should show a high degree of aggregation on the same factor. High convergent and discriminant validity is indicated if AVE (average variance extracted) > 0.5 and CR (composite reliability) > 0.7

Table S4 Convergent validity (N = 200)

Factor	item	Estimate	CR	AVE
cognition	a8	0.857	0.94	0.663
	a7	0.854		
	a6	0.85		
	a5	0.907		
	a4	0.755		
	a3	0.793		
	a2	0.73		
	a1	0.752		
transformation	b3	0.947	0.9392	0.834
	b2	0.941		
	b1	0.855		
implementation	c9	0.672	0.942	0.646
	c8	0.745		
	c7	0.859		
	c6	0.874		
	c5	0.904		
	c4	0.876		
	c3	0.725		
	c2	0.751		
	c1	0.792		
knowledge	d6	0.803	0.8778	0.560
	d5	0.882		
	d4	0.895		
	d3	0.467		
	d2	0.826		
	d1	0.483		
personal	e4	0.761	0.7784	0.471
	e3	0.753		
	e2	0.651		
	e1	0.561		

The difference in scores between the high and low groups in this study was significant ($p < 0.001$), indicating satisfactory discriminant validity of the 30 items of the scale. The criterion for the discriminant validity of factors is that the r^2 between each factor and the other factors is less than the AVE for that factor. The difference in scores between the high and low groups in this study was significant ($p < 0.001$), indicating satisfactory discriminant validity of the 26 items of the scale. The criterion for the discriminant validity of factors is that the r2 between each factor and the other factors is less than the AVE for that factor. In this study, the square roots of the AVE values of the five factors were 0.814, 0.903, 0.803, 0.748, and 0.686, respectively, all of which

were greater than their own correlation coefficients with other factors.

Table S5 Distinguishing validity: pearson correlation and AVE square root values (N = 200)

Factor	cognition	transformation	implementation	knowledge	personal
cognition	0.814				
transformation	0.698**	0.903			
implementation	0.735**	0.787**	0.803		
knowledge	0.663**	0.681**	0.730**	0.748	
personal	0.521**	0.585**	0.634**	0.527**	0.686

**P<0.01

Square root of the AVE value

Table S6 Correlation coefficients between factors and between aggregate tables (N = 200)

Factor	cognition	transformation	implementation	knowledge	personal	total
cognition	1	0.698**	0.735**	0.663**	0.521**	0.878**
transformation	0.698**	1	0.787**	0.681**	0.585**	0.858**
implementation	0.735**	0.787**	1	0.730	0.634	0.935
knowledge	0.663**	0.681**	0.730**	1	0.527**	0.844**
personal	0.521**	0.585**	0.634**	0.527**	1	0.715**
total	0.878**	0.858**	0.935**	0.844**	0.715**	1

**P<0.01
